# A fair lexical decision task for monolingual and multilingual Spanish-speakers

**DOI:** 10.3389/fpsyg.2026.1826045

**Published:** 2026-06-23

**Authors:** Julian M. Siebert, Mia Jimenez, Wanjing Anya Ma, Carrie Townley-Flores, Ana Saavedra, Jason D. Yeatman

**Affiliations:** 1 Graduate School of Education, Stanford University, Stanford, CA, United States; 2 University of California, San Francisco, San Francisco, CA, United States; 3 Division of Developmental-Behavioral Pediatrics, Stanford University School of Medicine, Stanford, CA, United States

**Keywords:** bilingualism, lexical decision task, multilingualism, reading assessment, Spanish

## Abstract

This study describes the development and validation of ROAR Palabra, a novel Spanish lexical decision task designed for use with both Spanish-speaking children and Spanish-English bilinguals. This self-administered task requires students to decide whether a string of letters presented on the screen is a real word in Spanish. While there is evidence that scores on English lexical decision tasks are highly predictive of performance on conventional (time- and resource-intensive) word reading assessments in English, we explore whether this holds in Spanish, which has a much more transparent orthography. The specific goals are (i) to create a linguistically fair task using item-response theory and (ii) to evaluate whether such task can serve as a reliable proxy for conventional word reading measures, offering a quick and easy-to-administer tool for assessing reading skills across linguistic and cultural contexts. Results demonstrated strong correlations between performance on ROAR Palabra and standardized word reading assessments such as the Woodcock-Muñoz Batería IV, suggesting its effectiveness as a substitute measure. Notably, the task was sensitive to differences in language proficiency across both monolingual and multilingual groups, reflecting expected developmental and environmental influences. While not specifically designed for the comparisons between monolingual and multilingual populations, the findings underscore the potential of this task as a versatile and culturally adaptable tool for reading assessments in different Spanish-speaking and bilingual contexts.

## Introduction

Reading proficiency is a cornerstone of academic achievement and cognitive development, influencing individuals’ ability to engage with and comprehend written information across various contexts. Therefore, learning to read is one of the main goals in early elementary school education (Catts, 2021). Efficient and equitable assessment of reading skills is essential for the early identification of struggling students so that instruction can be tailored to each student’s unique needs. However, traditional reading assessments are often time-consuming, resource-intensive, and may lack cultural and linguistic adaptability, particularly when applied to linguistically diverse populations such as multilingual individuals (Solano-Flores, 2016)—a population that is understudied and often misconceptualized (Bialystok, 2017; Cummins, 2000; Grosjean, 2008). In response to these challenges, there is a growing need for innovative assessment tools that are both reliable and scalable, as well as capable of functioning effectively across different linguistic settings.

In English, lexical decision tasks (LDTs) such as ROAR Word (Yeatman et al., 2021), which have a long tradition in cognitive science research (Balota et al., 2007), have been found to be efficient and reliable predictors of reading performance, correlating strongly with traditional assessments of word reading (Yeatman et al., 2021). In this study, we extend this approach to Spanish: We describe the development of ROAR Palabra, a novel self-administered Spanish LDT and investigate its relationship to traditional proctored assessment of Spanish word reading. Importantly, the task is designed around the linguistic diversity of both monolingual Spanish speakers in Latin America and Spanish-English bilinguals in the United States (US).

## Lexical decision tasks

LDTs require participants to determine whether a string of letters constitutes a real word or a pseudoword, a process that necessitates both decoding skills and lexical retrieval. LDTs are particularly effective in assessing word decoding and are widely used in psycholinguistic research to study word recognition and lexical access (Balota et al., 2006; Katz et al., 2012; Keuleers and Brysbaert, 2011). The literature largely agrees on the assumption that the underlying visual word recognition processes of a two-alternative forced-choice (2AFC) design in LDTs mirror the cognitive processes at play during other word recognition tasks, such as single-word reading out loud (Balota et al., 2006; Seidenberg and McClelland, 1989).

LDTs can serve different theoretical purposes depending on their design and implementation. Some lexical decision tasks are designed to measure *vocabulary size* while others are intended to assess *automatic word recognition* (also referred to as sight word reading). Vocabulary-focused LDTs typically present words without time constraints, allowing participants sufficient time to access semantic knowledge and make deliberate lexical judgments (Keuleers et al., 2015). In contrast, LDTs designed to assess automatic word recognition (like ROAR Palabra) employ brief stimulus presentations and emphasize response speed, targeting the rapid, effortless identification that characterizes fluent sight word reading (Coltheart et al., 2001).

This distinction becomes particularly important when considering the developmental progression of reading skills. Readers progress through distinct phases: from pre-alphabetic recognition based on visual cues, through partial alphabetic decoding of salient letter-sound relationships, to full alphabetic decoding, and finally to consolidated alphabetic processing where readers can recognize words instantly and automatically (Ehri, 1995, 2005). The transition from effortful decoding to automatic sight word recognition represents a critical milestone in reading development, as it frees cognitive resources for higher-level comprehension processes (Share, 1995).

The speed of word recognition is central to distinguishing between these processes. Skilled readers can recognize familiar words within 200–250 milliseconds (Seidenberg and McClelland, 1989), a timeframe that precludes deliberate phonological decoding. LDTs designed to capture this automatic recognition must therefore employ presentation times that require rapid lexical access rather than allowing time for sequential decoding strategies. ROAR Palabra employs stimulus presentations for 350 milliseconds (ms) to target this automatic recognition process rather than deliberate vocabulary knowledge assessment.

By measuring the accuracy and/or speed of responses on LDTs, such tasks can provide valuable insights into a student’s reading development and the degree to which word recognition has become automatized. The simplicity and efficiency of the task (easy and short administration) offers a quick and cost-effective means of gauging reading proficiency that can serve as a proxy for more comprehensive, individually-administered assessments. LDTs’ utility in predicting performance on traditional reading measures has been well-documented, particularly in languages with complex orthographies like English. Yeatman et al. (2021) show that students’ scores on the English ROAR Word are highly correlated (*r* = 0.94) with their scores on the Woodcock-Johnson Letter-word Identification subtask.

LDTs also offer a number of practical advantages over conventional word reading tasks, such as the Woodcock-Muñoz Letter-word Identification subtest (Woodcock et al., 2019). For one, their easy 2AFC design allows for objective, automatic, and immediate scoring without the need for verbalization. Because each item takes less than a second, the task can be completed within a few minutes and is amenable to computer adaptive testing (Ma et al., 2023). Last, the silent nature of a LDT allows for completion in a large group setting (e.g., classroom), which translates to less loss of instructional time and lower demands on resources in educational settings.

## Reading in transparent versus opaque orthographies

Orthographic transparency refers to the level of consistency in the phoneme-graphene correspondence in a language’s writing system. It influences the cognitive processes involved in word recognition and reading fluency and, therefore, is a crucial consideration when developing any reading assessment. Spanish is characterized by a highly transparent (shallow) orthography, where most letters or combinations of letters reliably represent specific sounds. In contrast, opaque orthographies like English exhibit numerous irregular spellings and inconsistent phoneme-grapheme mappings, which makes the process of reading—and learning to read— more complicated and thus extends the length and amount of instruction required to achieve mastery (Seymour et al., 2003; Ziegler and Goswami, 2005).

The application of LDTs in languages with transparent orthographies presents unique opportunities and challenges. The transparency of the Spanish language facilitates the ease of acquisition of foundational reading skills. Research indicates that Spanish-speaking children typically develop phonological skills more rapidly than same-aged peers learning to read in less transparent languages (Ziegler et al., 2009). This accelerated acquisition of letter-sound correspondence and phonological awareness, in turn, allows for an earlier focal shift toward decoding skills and reading fluency (Aguasvivas et al., 2020).

Decoding skills allow readers to translate written text into spoken language based on acquired letter-sound correspondence. Over time, decoding becomes automatized allowing for rapid and accurate word recognition. Automated word-level decoding skills facilitate the reading of individual words and are precursors to sentence-level reading efficiency and comprehension (Ehri, 2005; Perfetti, 1985). The relatively straightforward syllable structure of Roman languages, characterized by predominantly open syllables (CV-CV) and limited consonant clusters, facilitates more efficient grapheme-phoneme mapping and thus enhances the ease of decoding for readers (Seymour et al., 2003). Therefore, in transparent orthographies where decoding is relatively straightforward, word recognition (alongside reading fluency) becomes one of the primary early indicators of reading proficiency.

Then, the ease of grapheme-phoneme correspondence in Spanish may enhance the utility of LDTs in assessing word recognition and reading fluency, due to the relatively low decoding demand (Seymour et al., 2003). However, the higher transparency also means that LDTs must be carefully designed to differentiate between varying levels of lexical access and processing speed among different proficiency levels (Vega-Mendoza et al., 2015). Generally, research is sparse on the use of LDTs in Spanish (Aguasvivas et al., 2020), particularly for the assessment of multilingual learners.

### Assessing multilingual individuals

More than half of the global population is believed to be multilingual (Grosjean, 2010). In the US, about 10% of K-12 students speak a language other than English as their first language; in California this holds true for about 20% of the population (California Department of Education, 2023; National Center on Improving Literacy, 2023). The development of most psychological and educational assessments, however, continues to largely operate from within a monolingual English mindset. Decisions based on inappropriate assessment choice or interpretation of results can have drastic consequences and may result in multilinguals’ educational needs going unmet (Umansky, 2016). In this paper, we aim to shift this paradigm toward a more careful consideration of multilingual individuals in assessment development to ensure fairly comparable outcomes (Faulkner-Bond and Soland, 2020; Solano-Flores, 2023).

Multilingual individuals are a heterogeneous population with different levels of language proficiency and reading skills across their languages. They exhibit unique developmental trajectories that reflect differences in the amount and sequence of language acquistion, exposure, formal and informal learning environments, as well as sociocultural context (Solano-Flores, 2016; Surrain and Luk, 2019). Durán et al. (2024), for example, report that multilingual Spanish-English bilinguals with different levels of English proficiency show different levels of growth on various foundational reading skills in kindergarten and first grade, when assessed in English. Especially students with high levels of proficiency in their different languages (balanced multilinguals) are able to tap into all of their languages’ linguistic resources, which benefits their metalinguistic skills (e.g., phonological awareness) and supports cross-linguistic knowledge transfer (Barac et al., 2014).

The effect of concurrent or sequential exposure to multiple languages and linguistic environments can allow for cross-linguistic transfer, where skills developed in one language influence the acquisition and proficiency of another (Cummins, 2000). In the context of reading, individuals may also transfer phonological awareness and decoding strategies from their dominant language to their second language, enhancing their reading development in both. The nature and extent of this transfer can vary depending on factors such as language similarity, proficiency levels, and the context of language use. For Spanish-English bilinguals, the transparent orthography of Spanish may facilitate the transfer of decoding skills to English, while the less transparent English orthography may, in turn, influence reading strategies employed in Spanish (Bialystok, 2017).

Understanding the dynamics of reading development is essential for developing assessments that accurately reflect the reading abilities of multilingual individuals. The variability in developmental trajectories of multilingual readers means there is a need for assessment tools that are sensitive to these learning differences and can provide equitable measures of reading proficiency across both monolingual and multilingual populations. Unfortunately, most readily available reading assessments were designed with monolingual English populations in mind—as well as in the calibration and norming samples. These assessments do not adequately account for the heterogeneity and complexities of the multilingual experience, therefore often underestimating multilingual individuals’ true linguistic abilities (Bialystok, 2001; Luk and Bialystok, 2013; Solano-Flores et al., 2009; Solano-Flores and Hakuta, 2017).

Linguistically fair assessment means for a measure to produce equally valid and accurate results for test-takers with different linguistic backgrounds (e.g., first languages, different levels of proficiency of the same language, etc.), but equal levels of the latent trait of interest. In other words, a linguistically fair measure for use with mono- and multilingual individuals must not be biased in favor of or against those test-takers that are multilingual. This becomes a difficult endeavor when the trait to be assessed is a language-related construct, such as in the case of an LDT.

For LDTs, when used with multilingual populations, this means that they must account for cross-linguistic influences, cultural influences, and varying degrees of language dominance to ensure accurate assessment. Thus far, Spanish LDTs were successfully used with Spanish-English bilinguals in a sample of tertiary students with high and low English proficiency (Fairclough, 2011). Moreover, Aguasvivas et al. (2020), using LDTs as a measure of Spanish vocabulary, also found no statistically significant changes between monolingual and bilingual tertiary students. We are not aware of any study that examined this in younger populations of multilinguals, or as measures of decoding.

## Research questions and aims

1. The first goal is to develop a reliable Spanish lexical decision task. Specifically, we aim to build the ROAR Palabra, a self-administered Spanish lexical decision task use with both monolingual children in Colombia and multilingual children in the United States.
2. Second, we investigate the efficacy of such a task for use as a proxy for Spanish single word reading skills, as measured by conventional, resource-intensive, proctored assessments.

## Methods

### Participants

Our sample (*N* = 6,427) comprises children from two locations: We recruited a mostly monolingual Spanish-speaking sample from Bogotá, Colombia, (*n* = 5,582), as well as a sample of Spanish-English multilingual children from across the United States (US), mostly from California (*n* = 845). The Colombian sample comprises students in grades 1 to 11 at two public schools and one concession school located in Bogotá, Colombia. Concession schools (colegios en concesión) were first launched in Bogotá in 2000; the government contracts private operators to run these schools. Concession school students, on overage, receive higher scores on national standardized tests (pruebas Saber) relative to students in other public schools. Our sample comes from a schools located in two different low-income neighborhoods of Bogotá. Additional demographic information was limited.

Table 1 provides an overview of the US sub-sample’s demographics. The majority of this sub-sample comes from a Northern Californian school district with a large proportion of students classified as English learners and an intentional focus on multilingual learning, manifesting in, for example, the provision of dual-language immersion programs. At other US sites, selection of students happened at the school’s discretion, though they mostly also selected students classified as English learners or used teacher judgment.

**Table 1:** United States sub-sample’s demographic characteristics.

Characteristic	G1 *N* = 327^1^	G2 *N* = 329^1^	G3+ *N* = 189^1^
Location (within US)			
California	327 (100%)	329 (100%)	51 (27%)
Other	0 (0%)	0 (0%)	138 (73%)
Gender			
Female	159 (51%)	129 (40%)	1 (50%)
Male	150 (49%)	193 (60%)	1 (50%)
Unknown	18	7	187
English proficiency designation			
English Learner	202 (65%)	211 (66%)	0 (0%)
English-only	73 (24%)	74 (23%)	1 (50%)
English-proficient	34 (11%)	36 (11%)	1 (50%)
Unknown	18	8	187
Free or reduced-price lunch eligibility			
Eligible	218 (70%)	235 (72%)	1 (25%)
Not eligible	94 (30%)	90 (28%)	3 (75%)
Unknown	15	4	185

^1^*n* (%).

### Measures

#### ROAR Palabra

ROAR Palabra is a silent Spanish lexical decision task, which requires test-takers to decide whether an item flashing on the screen for 350 ms is a real Spanish word versus a made up word (pseudoword) and to respond via pressing a button on the keyboard/touchscreen. There is no limit on the response time but the item is only presented for 350 ms. It is an online assessment instrument, developed to accurately measure students’ word reading ability in a time- and cost-efficient manner, doing away with the necessity for one-on-one assessments by trained assessment experts. It is modeled on ROAR-Word (Yeatman et al., 2021), which has shown to have high internal consistency reliability (*r* = 0.95) and scores on which highly correlate with Woodcock-Johnson letter-word identification scores (*r* = 0.94).

Importantly, ROAR Palabra is explicitly *not* a translation of ROAR-Word—as a simple translation does not create equivalent versions of the same test (Solano-Flores et al., 2009). In contrast to many other non-English measures, we started the development process from a Spanish perspective: We created an initial list of stimuli by prompting ChatGPT to produce a list of Spanish words that met specific criteria: (i) they must be high frequency words appearing in the top 5,000 most common words according to frequency databases such as SUBTLEX-ESP (Cuetos et al., 2011) and Davies (Davies, 2006); (ii) the list must contain words normally acquired by children in the course of their elementary and middle school years, or aged 6–14; (iii) recognized across Spanish-speaking regions in the Americas; and (iv) occurring in all major varieties of Spanish without regional restrictions. The frequency criterion was operationalized as words with subtitle frequencies ≥10 per million words in SUBTLEX-ESP, corresponding to words that appear regularly in everyday discourse and are likely to be familiar to developing readers.

We then used the Wuggy algorithm (Keuleers and Brysbaert, 2010) to create matching, word-like pseudowords—stimuli conforming to Spanish orthographic rules and matching the real word list in terms of word length, letter-transition frequencies, and orthographic neighborhood size, measured using Orthographic Levenshtein Distance (Yarkoni et al., 2008). Five native Spanish speakers representing different varieties of Spanish (from Colombia, Ecuador, Mexico, Spain, and United States) then independently reviewed both the real words and pseudowords. The expert reviewers assessed each item for: (a) cross-regional familiarity and appropriateness, (b) frequency of use in their respective dialects, (c) age-appropriateness for elementary and middle school students, and (d) absence of inappropriate or offensive meanings.

Items were flagged for removal if they had frequency ratings below 10 per million in any major Spanish frequency database (Cuetos et al., 2011; Davies, 2006), were judged unfamiliar by any reviewer, or carried inappropriate connotations in any Spanish variety. Additional linguistic properties were considered during item selection, including word length (4–12 letters), syllabic structure (predominantly CV patterns typical of Spanish), and phonological complexity. With the Spanish-English bilingual context in the US in mind, we also removed generated pseudowords that were real words in English, ensuring that bilingual participants would not be disadvantaged by cross-linguistic interference.

This process resulted in an initial item bank with 378 items (that is 189 real words and 189 matched pseudowords). To keep administration time reasonable, we selected 70 items (35 real and 35 pseudowords), as part of the *core corpus*. These items were representative of the overall item bank in terms of item characteristics such as length and frequency, spanning a broad range (see Appendix A for detailed item characteristics). We refer to the remaining 308 items as the *extended corpus*. Every test-taker responded to *all* core-corpus items, as well as random sample of items selected from the extended corpus. This ensured that we collected enough reponses on the same set of items to carry out the necessary analyses. Appendix Figure A1 shows the distribution of item characteristics (e.g., word length, frequency) for the initial list of items.

ROAR Palabra can be accessed by filling out a Partner Interest Form at: roar.stanford.edu.

#### Woodcock-Muñoz Batería IV

To assess the degree to which performance on the silent, self-administered ROAR Palabra can function as a proxy for conventional, individually administered word- and nonword reading, we used two subtests of the WM (Woodcock et al., 2019)—a Spanish parallel of the Woodcock-Johnson IV (Schrank et al., 2014):

1. The *identificación de letras y palabras* (letter-word identification; WM-LWID) test, measuring children’s oral reading ability by having them read out aloud increasingly difficult words.
2. The *análisis de palabras* (word attack; WM-WA) test, requiring children to read increasingly complex nonsense word out aloud, thereby tapping into their phonics and decoding skills.

Both tasks are scored for pronunciation accuracy by trained test-administrators following the guidelines of the scoring manual. The age-standardized scores of both the WM-LWID and WM-WA can be aggregated to provide a basic reading skills (WM-BRS) composite score.

### Procedures

In both settings, we worked closely with school partners in conducting this study. School partners provided information about the study to parents or guardians, who then had the opportunity to opt out if they preferred for their children to not participate in the study. All students saw assent forms on the screen before beginning ROAR-Palabra and the researchers ascertained verbal assent before completing WM testing. All protocols were approved by the Institutional Review Board at Stanford University.

In Colombia, we trained a group of 20 field assistants and a field coordinator for the administration of both ROAR Palabra and as well as both WM subtests. All students at participating schools took ROAR Palabra in the computer rooms of the school, unless their parents had opted out. Then, we randomly selected approximately 25% of those who had completed ROAR Palabra (balanced across grade levels) to also complete the WM-LWID and WM-WA. For this, students were taken to a dedicated space in the school library or a multi-purpose room and completed the task on a laptop with a proctor—either in-person or on a laptop with headphones, connected to a proctor via a video-conferencing software. Scores obtained this way were double-scored by experienced WM administrators.

In the US, exact study procedures varied by school district. In most instances, schools made laptops or tablets available to students and tested whole classrooms at a time. Research coordinators provided training and (on-site) support before and during the administration periods.

### Analysis

#### Item-response theory calibration

In addressing the first aim of the study—building the first version of ROAR Palabra—we undertook several steps to obtain a final item-response theory (IRT) model. Prior to doing model building, we (i) obtained raw difficulty estimates for each item (proportion of all respondents that responded correctly to a given item), (ii) filtered responses based on children’s median response times (< 450 ms) and correctness rate (< 65%) to exclude random guessers, (iii) excluded items with low (< 0.10) point-biserial correlations to ROAR Palabra (core-corpus items only) totals and WM-LWID totals, and (iv) excluded items with suboptimal item in- and out-fit (< 0.60 or > 1.40) within the core corpus. We applied these criteria separately to both the Colombian and US sub-samples, so that characteristics of both populations are represented in the final item selection.

Using the responses retained after excluding rapid guessers and with those core-corpus items surviving the point-biserial correlation exclusion criteria, we iteratively fit a 1PL model and excluded all items with poor fit until we obtained a stable model of the form

$$P(X_{i}=1|\theta )=0.5+0.5\frac{\exp(\theta -b_{i})}{1+\exp(\theta -b_{i})}\;\;\;\;$$ (1)

Where $P(X_{i}=1|\theta )$ is the probability of a correct response given item i ’s difficulty level, $b_{i}$ , which measured on the same scale as the respondent’s ability, θ . Given the 2AFC task design, we imposed a 0.50 lower bound on the probability of a correct response (guessing parameter). We used the mirt package (Chalmers, 2012) for R (R Core Team, 2018) for all IRT analyses and calculated theta scores using the default and expected a-posteriori (EAP) estimator.

We then fit a two-parameter (2PL) model, in order to be able to evaluate item discrimination parameters ( α ). While not used in the final theta estimation, α denotes the steepness of the slope of the item characteristic curve, indicates the steepness of the slope of the item characteristic curve and is an indicator of how well that item discriminates between two respondents whose ability levels are around the item’s difficulty. This provides important information about the item’s quality and usefulness in the final measure. Typically, the range of the α parameter is from 0 to 3, with an α<.50 indicating less productive measurement.

Next, we fit a final 1PL model using Equation 1, which is the model used to obtain ROAR Palabra scores. This time, we included all those items in the *entire* item bank (core and extended corpora) that survived the exclusion criteria outlined above. We fixed the scale using the item parameters obtained in the model for the core-corpus items and only estimated item parameters for the extended-corpus items. Again, we fit a 2PL model for the purpose of obtaining item discrimination parameters.

#### Reliability

Following this, we evaluated the reliability of the final (1PL) model. Given that ROAR Palabra is a fixed-length task scored using a 1PL model, the appropriate reliability metric is empirical reliability ( $\rho_{xx^{\prime }}$ ), estimated using Equation 2.

$${\hat{\rho }}_{xx^{\prime }}=\frac{\hat{\mathrm{VAR}}(\hat{\theta })}{\hat{\mathrm{VAR}}(\hat{\theta })+\hat{\mathrm{SE}}{\left(\hat{\theta }\right)}^{2}}\;\;\;\;$$ (2)

#### Parameter invariance

Next, we assessed the final model’s parameter invariance—that is, we checked whether item difficulty and discrimination parameters are significantly different in the two sub-samples. To do so, we compared item parameters from a jointly calibrated 1PL model to parameters obtained from 1PL models fit separately for each sub-sample, as well as parameters from the two separately calibrated models. Because the two sub-samples cover very different grade ranges, we conducted the parameter invariance analysis on a separate set of models using only data from respondents in the overlapping grade range.

#### Criterion validity evidence

Finally, we assessed ROAR Palabra’s criterion validity. For this we used the ROAR Palabra theta scores obtained using the final 1PL model, as well as Colombian students’ raw scores on the WM-LWID, WM-WA, and WM-BRS. We correlated students’ observed WM scores with predicted WM scores obtained from a generalized additive model with a smooth function on ROAR Palabra theta scores. Finally, given the sub-samples different grade ranges, we repeated all analysis steps using only the overlapping grades as a sensitivity analysis in the Appendix.

The code used to run the analyses and to generate this manuscript is available in this OSF Project: https://osf.io/rhu3w/.

## Results

### Item responses

For the 70 core-corpus items, we have 5,582 observations per item for the Colombia sub-sample and 845 per item for the US sub-sample. For the extended corpus items, while we observe sufficiently large numbers for the purpose of item calibration for the Colombia sub-sample, the response counts from the US are too small to reliably calibrate an IRT model to the US sub-sample. Therefore, we refrain from comparing performance on the extended corpus and restrict our detailed analyses and item parameter estimation to the core corpus. Afterwards, we refit the model with the extended-corpus items while holding the core-corpus items’ parameters fixed, so that the extended-corpus corpus items are calibrated to the same measurement scale that was defined based on the detailed analysis of the core corpus item bank.

### Sample performance and median response times

**Figure 1:**
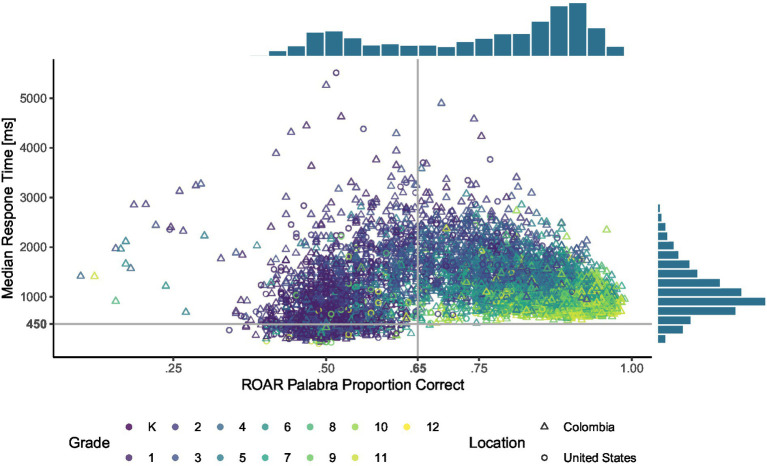
Median response time as a function of raw (proportion correct) score on ROAR Palabra.

Figure 1 shows median response times as a function of raw scores (calculated as the percentage of correct responses for each student), disagreggated by grade. Barely any of the students performing above chance exhibit median response times < 450 ms. At the same time, students with extremely fast response times (<450 ms) perform around the chance level, which is likely indicative of rapid guessing. The bimodality of the raw score distribution can be explained by the large grade range, which includes both children still learning how to read words, as well as high schoolers who largely mastered that skill. Indeed, Appendix Figure C1 shows the same analysis for only those grades (1 and 2) that are represented in both samples; the overall patterns are very similar and no difference based on study location is observed.

### Item properties

**Figure 2:**
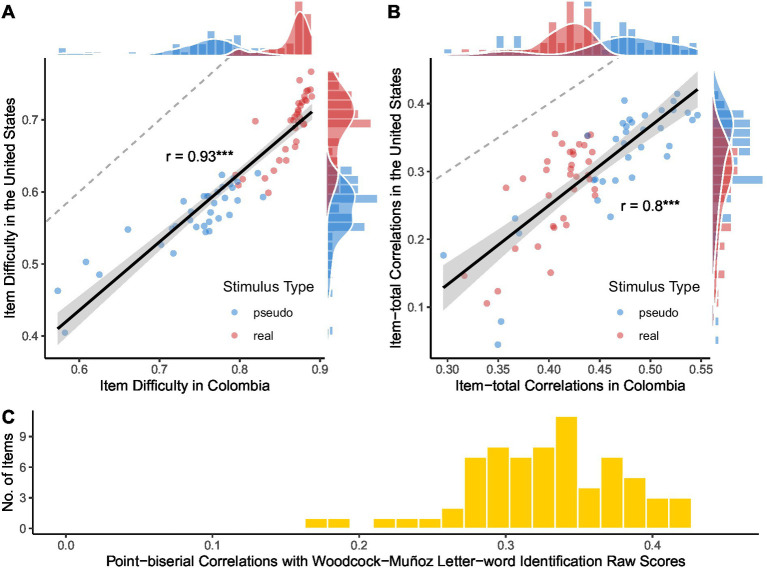
ROAR Palabra item properties with item difficulty (proportion correct) distribution in panel **(A)**, item-total (point-biserial) correlations in panel **(B)**, and item-WM-LWID (point-biserial) correlations in panel **(C)** (Colombian subsample only), for core-corpus items.

Figure 2A shows that, for both sub-samples, raw item difficulty follows a bimodal distribution with real words (right peak) being easier than pseudowords (left peak). The shift in positions of the distributions indicates that, on average, items are easier for the Colombian students (which cover a much larger grade range). The very high correlation between item difficulty in the two sub-samples (*r* = 0.93) indicates that the relative positions of items on item difficulty distribution are very similar in both sub-samples. This finding also holds in the separate analysis for grades 1 and 2 only (Appendix Figure C2A).

Point-biserial correlations between students’ responses to a given ROAR Palabra item and their raw (proportion correct) ROAR Palabra score are indicators of the degree to which an individual item taps into the same construct that is measured by the overall scale. Figure 2B shows that ROAR Palabra forms a coherent scale; within the Colombian sub-sample, all correlations are > 0.20, within the US sub-sample, the majority is, too.

Further, point-biserial correlations between students’ responses to individual ROAR Palabra items and their WM-LWID raw score indicate to what extent each item is related to the construct of word reading. Figure 2C shows the distribution of these point-biserial correlations. While two real words show very low (< 0.10) point-biserial correlations, the majority of items fall into an acceptable range.

Overall correlations of item parameters between the two study locations show similar trends when analyzed separately for the lower grades only. Appendix Figures C2A–C report repetitions of the analyses described here for the subs-sample of students in grades 1 and 2 only.

### IRT model building

We started the model building process with the 70 core-corpus items, because we had sufficiently large numbers of observations in both contexts. For the extended-corpus items, of which test-takers only saw random selection of 30 out of the 308 items, response counts in the US sub-sample were low. Therefore, we add those items at a later stage, after the calibration of a measurement model based on the core corpus. Prior to the estimation of an IRT model, we carried out four item selection steps as follows:

#### Participant exclusion criteria: median response time

We excluded data from participants whose response behavior was indicative of random guessing or clicking through the task without a serious attempt at the task. This was operationalized as a median response time < 450 ms and a score of < 65% correct. This resulted in an exclusion of 6.94% (*n = 446*) of participants in total, 5.71% (*n = 319*) of Colombian participants and 15.03% (*n = 127*) of participants from the US. Item counts are not affected by this exclusion criterion.

#### Item exclusion criteria

##### Criterion 1: item-total correlations

As a first step toward ensuring we are measuring a single and coherent construct, we proceeded to eliminate all those items that exhibited point-biserial correlations with the total raw task score (proportion correct) of less than 0.10—a very lenient threshold. In other words, this means removing those items whose response patterns are unrelated to the overall proportion correct scores. To account for both contexts, we applied this criterion separately to the Colombian and the US sub-sample. This resulted in the exclusion of 3% of items (2 items; 0 real words and 2 pseudowords) based on data from the US. The pseudowords excluded were *cumpleapos, estudionte*. No items had to be excluded based on data from Colombia.

##### Criterion 2: item-WM correlations (Colombia only)

Next, we computed correlations to WM-LWID raw scores and had planned to exclude all items that exhibited point-biserial correlations < 0.10. No items were flagged in this stage.

##### Criterion 3: item-fit

Next, we iteratively fit a 1PL model and assessed item fit. A lenient range for good item in-fit and out-fit parameters is 0.60–1.40. Only 2 items fell outside this range.

#### Core model

After applying the four above criteria, the resultant core corpus item pool contains a total of 66 items (33 real words and 33 pseudowords). None of these items exhibited low ( α<0.50 ) item discrimination parameters. Moreover, as was the case with the raw score, the distributions of theta scores show differences between the US and Colombian sub-samples, though these disappear when filtered to only draw on the earlier grades.

#### Final model

**Figure 3:**
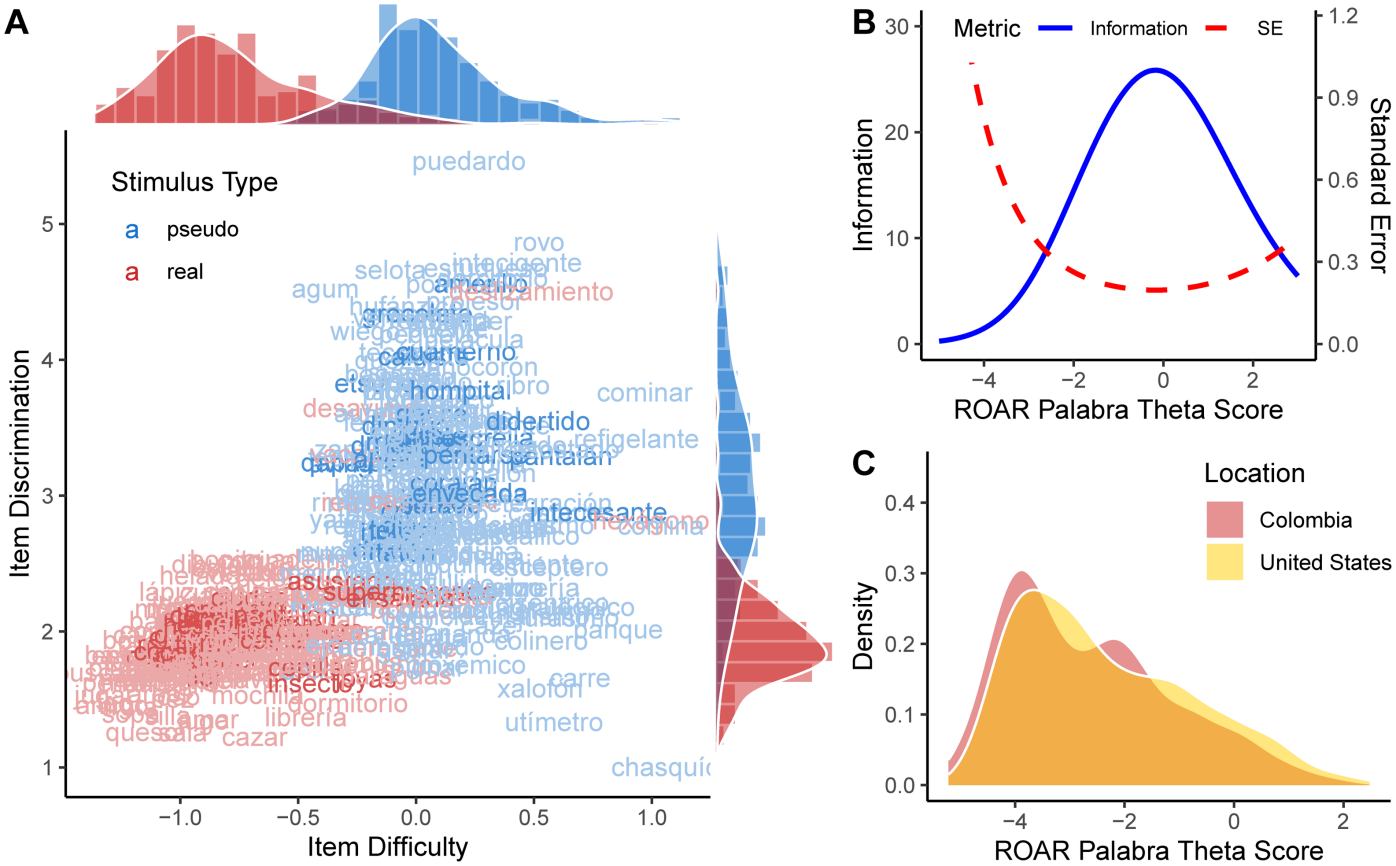
Summary of final ROAR Palabra item-response theory model, showing the bivariate distribution of item difficulty and discrimination **(A)**, the test information curve with the associated standard error (SE) **(B)**, and the distribution of theta scores for the overlapping grade range (grades 1 and 2) by location **(C)**.

Before adding the items from the extended corpus to the core measurement model, we subjected them to the same four criteria we used to prune the core corpus item pool. We then fixed the core-corpus items’ parameters and refit a 1PL model with guessing parameter to estimate the new (extended-corpus) items’ difficulty parameters. We also reran the 2PL model to obtain item discrimination estimates, this time only estimating the new items’ discrimination parameters. Figure 3 summarizes the final models’ characteristics and performance: Figure 3A shows the resultant distribution of item difficulty and discrimination parameters; Figure 3B shows the test information curve and the associated standard error; and Figure 3C shows the distribution of theta scores by location for grades 1 and 2 (which can be compared fairly, as they are covered in both sub-samples), which closely mirror the distributions obtained using the core model. Appendix Figure B1 shows the theta score distribution for all grades.

##### Reliability

We estimated empirical reliability for the final model (*n* = 5,981), comprising both core- and extended-corpus items, using Equation 2. Overall reliability is high, with $\rho_{xx^{\prime }}$ = 0.938, as are reliability estimates for Colombia ( $\rho_{xx^{\prime }}$ = 0.887, *n* = 718) and the United States ( $\rho_{xx^{\prime }}$ = 0.936, *n* = 5,263), separately. Table 2 shows empirical reliability estimates by grade, drawing on both the Colombian and US data.

**Table 2:** ROAR Palabra empirical reliability by grade (Colombia and US).

Statistic	All	Gr 1	Gr 2	Gr 3	Gr 4	Gr 5	Gr 6	Gr 7	Gr 8	Gr 9	Gr 10	Gr 11
*n*	5,977	643	799	532	519	580	615	542	428	466	430	423
*r*	0.938	0.705	0.877	0.918	0.919	0.910	0.890	0.860	0.849	0.865	0.797	0.839

##### Parameter invariance

**Figure 4:**
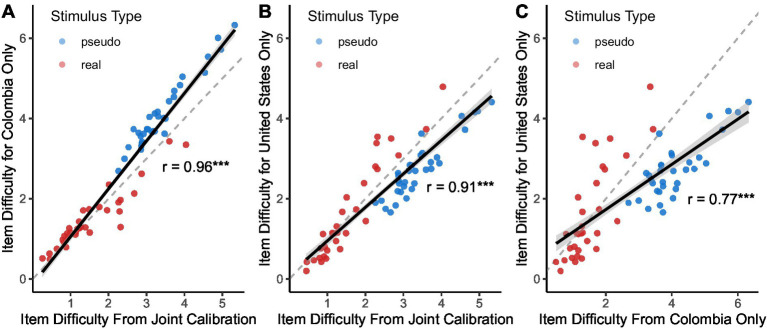
Parameter invariance analysis for 1-parameter logistic model (grades 1 and 2 only) in the form of correlations between jointly and separately calibrated item parameters for Colombian sub-sample **(A)** and United States sub-sample **(B)**, as well as between the two separately calibrated models **(C)**.

Next, we assessed parameter invariance. To account for the difference in grade ranges, we fit another set of 1PL models using only data from respondents in those grades represented in both samples (grades 1 and 2). We compared item parameters of a jointly calibrated IRT model to parameters for separately calibrated models, as well as correlations between parameters of the two separately calibrated models. Figure 4 shows the resultant correlations. Both sub-samples’ item parameters are very highly correlated with those obtained from a joint calibration. The correlation between separately calibrated US and Colombian parameters, though somewhat lower, still suggests that parameters are similar in both contexts. Here, the lexicality effect, with pseudowords being easier for the Colombian subsample, warrants further investigation.

### Validity evidence

**Figure 5:**
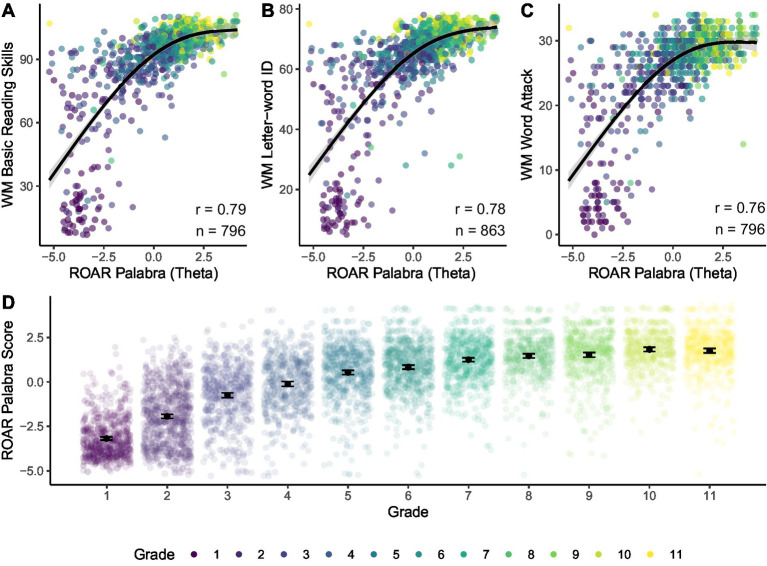
Validity evidence for ROAR Palabra by means of correlations between ROAR Palabra theta scores and Woodcock-Muñoz basic reading skills **(A)**, Letter-Word Identification **(B)**, and Word Attack **(C)** raw scores, as well as cross-sectional growth across grades on ROAR Palabra **(D)** for the Colombian sub-sample.

These analyses draw on a sub-set of the Colombian sub-sample. To assess whether ROAR Palabra scores can be used of indicators of word reading performance, we correlated students’ ROAR Palabra theta scores (obtained from the final model) with their scores on the WM Basic Reading Skills, WM-LWID and WM-WA scores (Figures 5A–C, respectively).

Cross-sectional growth patterns can provide additional validity evidence. As children progress through the grades, their score on lexical decision tasks is reasonably expected to increase, given the additional reading instruction and vocabulary expansion. Therefore, a good lexical decision task should produce higher scores for students in higher grades. Figure 5D shows that mean ROAR Palabra scores increase monotonically across grade levels.

## Discussion

This study investigated the feasibility of fairly using the same Spanish lexical decision task (LDT) with both monolingual Spanish-speaking and Spanish-English bilingual students, as well as such a task’s utility as a proxy for traditional, proctored word reading assessments. Specifically, we (i) successfully developed ROAR Palabra as a linguistically fair Spanish LDT with very similar item parameters and score distributions among US and Colombian first- and second-graders and (ii) showed its moderate to high correlations with the Woodcock-Muñoz Batería IV Letter-word Identification and Word Attack subtasks. Additionally, we found that—for both mono- and multilinguals—item difficulty is affected by lexicality.

### Lexicality affects item difficulty

The bimodal distributions of item difficulty and item discrimination (Figure 3A) suggest the presence of a lexicality effect. Items cluster closely together and the cluster of real-word items is less difficult and less discriminating than the cluster of pseudoword items. This means that correctly responding to real-word items (i.e., recognizing known words) is easier than correctly identifying pseudowords as such. Moreover, this tells us that pseudowords are more useful in telling apart high- from low-performers.

We are confident that this difference is largely based on lexicality. Given that we effectively controlled for length, phonotactic constraints, and orthographic neighborhood size when constructing the pseudoword-items by using the Wuggy algorithm (Keuleers and Brysbaert, 2010), these stimuli characteristics are similarly distributed within the real-word and pseudoword item groups. This lexicality effect holds true for both the mono- and the multilingual sample and is consistent across grades.

This clustering of items is not observed in other (less transparent) languages. In a very similar English task, Yeatman et al. (2021), for example, did not observe this pronounced difference in item difficulty and discrimination between real words and pseudowords. This lexicality effect likely generalizes to other languages with similarly transparent orthographies, but further research is needed to confirm this. In addition, this finding could be corroborated if this pattern holds even with a larger set of real-word items that are less common, longer, or more difficult due to some other item features.

### Decoding vs. vocabulary

One possible explanation for the presence of a lexicality effect in this Spanish LDT, but not in a comparable English task, is the lower decoding demand in Spanish (Ziegler and Goswami, 2005). Given that the task design sees the stimuli only appear briefly, efficient decoding is necessary in order to, in a second step, make a lexicality decision. In Spanish, due to its transparent orthography, the ability to correctly decode both words and pseudowords develops much faster than other reading skills, so that Spanish readers can successfully decode text before they can comprehend it (López-Escribano et al., 2013).

This would suggest that Spanish LDTs load less on efficient decoding skills, but are more directly affected by vocabulary size—or the ability to recognize a known word. Indeed, others have also used Spanish LDTs to assess vocabulary size (Aguasvivas et al., 2020). Our findings of lower correlations between ROAR Palabra scores and WM-LWID and WM-WA scores (both of which assess decoding), compared to parallel English analyses (Yeatman et al., 2021), supports this hypothesis.

### Linguistically fair assessment?

A linguistically fair assessment instrument produces equally accurate results for test-takers who have different linguistic backgrounds but equal levels of mastery of the target construct. In the present study, developmental stage can only be operationalized as grade level. This is caveated by the fact that students in Colombia receive formal education entirely in Spanish within a predominantly Spanish-speaking societal context, while US participants–—though classified as Spanish speakers—–experience varied linguistic environments, with some receiving instruction primarily in English while using Spanish predominantly at home. These different patterns of language exposure and academic instruction may result in distinct developmental trajectories for Spanish reading skills that cannot be adequately captured by grade-level comparisons alone. This raises the questions of whether we would expect all Spanish-speaking students at the same grade level to have achieved equivalent mastery of Spanish lexical decisions, regardless of amount and context of language experience, of linguistic environment, and of educational context.

Nonetheless, we presented evidence showing that ROAR Palabra item parameters are not statistically significantly different in the two contexts and that final theta scores for grades 1 and 2 are almost identically distributed in the two samples. This suggests that, when developed carefully, the same Spanish LDT may be used with both monolingual and multilingual Spanish-speaking first- and second-graders—at least in Colombia and the US. However, these findings should not be interpreted as evidence for equivalence in underlying Spanish reading ability across contexts. Rather, they suggest that the task functions similarly as a measurement instrument in both populations, which is a necessary but not sufficient condition for fair assessment.

This is in line with Aguasvivas et al. (2020), who—albeit in a Spanish-dominant context—found no statistically significant differences in vocabulary size assessed via LDT between monolingual and bilingual Spanish-speaking adults. In contrast, Izura et al. (2014) showed large difference between first- and second-language speakers of Spanish in favor of the former on the LexTALE-Esp., a similar task requiring the correct identification of presented stimuli as words or pseudowords. One possible explanation for this difference might be the different age groups for the sample; our sample of students in grades 1 and 2 is likely to largely comprise beginning decoders and that differences might only start manifesting later.

While we establish parameter invariance and obtain very similar score distributions for the grade range compared, these findings ought to be corroborated by additional bias analyses, especially for larger grade ranges. Our psychometric findings do not permit direct performance comparisons between Colombian monolingual and US bilingual students, as differences in performance may reflect legitimate variation in Spanish language development rather than measurement bias. The different linguistic and educational contexts may produce distinct developmental patterns that become more pronounced at higher grade levels, limiting the generalizability of our findings beyond early elementary grades.

Overall, these findings suggest that ROAR Palabra can function as a valid measurement instrument within each population (Colombian monolingual and US Spanish-speaking students), but direct cross-group comparisons should be avoided without appropriate normative data that accounts for linguistic background and educational context. Future research should develop population-specific norms and investigate developmental trajectories within each linguistic context to enable more nuanced interpretation of ROAR Palabra scores.

### Next, steps, and limitations

Our data is currently imbalanced in favor of the Colombian context. Therefore, we are currently collecting both ROAR Palabra and WM-LWID data from a more sizeable sample of Spanish-English bilinguals in higher grades in the US to further corroborate our claims. This will allow us to also provide criterion validity evidence for the US context. Even though the parameter invariance analysis suggested that item parameters are sufficiently similar in both the US and Colombian sub-samples, this will also need to be verified with a larger sample. Particularly for the items in the extended corpus, our observation counts in the US sub-sample are too low to inform item pruning. Additionally, the suggested lexicality effect warrants further investigation.

Furthermore, we plan to create new items—particularly more difficult real words—in order to increase the size of the item bank. A larger item bank with more well-performing items is necessary for an efficient use of a CAT algorithm. Though unlikely, once we will have collected more (US) data on the extended-corpus items, as well as on a set of additional items, we will have to revisit the IRT model and decide whether a re-calibration is warranted.

Additional analyses of interest relate to the features of individual items. Which features (length, cognates, age of acquisition, or position of the insertion, deletion, or substitution in the pseudoword creation process, etc.), in addition to stimulus type, make items easier or more difficult? Analyzing the characteristics of well-performing items will also help facilitate longer-term item bank development and inform other scholars developing Spanish reading measures.

Other important questions that need answering pertain to the generalizability of these findings. Do these findings hold true across other Spanish-speaking populations, or other school types or socioeconomic strata? It would also be particularly insightful to disaggregate the US data by instructional model, in order to check for effect of children’ language of instruction on ROAR Palabra scores and to reflect the notion that multilinguals are a heterogeneous population (Solano-Flores et al., 2009; Solano-Flores and Hakuta, 2017).

## Conclusion

Linguistically fair behavioral assessment for the growing multilingual population is a pressing global need. ROAR-Palabra is a reliable Spanish lexical decision task for use in both research and educational contexts, poised to respond to this. It was specifically developed for use with both first- and second-graders in monolingual Spanish-speaking settings, as well as in multilingual settings, such as with Spanish-speaking bilinguals in the US. Additionally, sensitivity to cross-sectional growth across grades and moderate to strong correlations with the Woodcock-Muñoz Basic Reading Skills cluster underpin its potential as an efficient proxy for otherwise time- and cost-intensive proctored reading assessments.

Beyond presenting psychometric evidence for this new task, however, this paper also functions as a blueprint for the development of linguistically fair assessment instruments. We argue that the development of a task intended for use in multilingual populations requires careful subgroup-specific analyses and, most importantly, the consideration of this population and its linguistic and cultural context through the *entire* development process. The former consideration manifests in subjecting the item pool to subgroup-specific item pruning and establishing parameter invariance between mono- and multilingual speakers. The latter substantiates in the representation of speakers of multiple varieties of Spanish, including bilingual Spanish-English speakers, in the developer team to ensure an unbiased item pool, as well as in the inclusion of multilingual speakers in the model calibration (and later norming) sample.

## Data Availability

The dataset for this study cannot be made publicly available due to agreements with the partner schools. Inquiries about the dataset can be directed to the corresponding author/s.
